# Diagnostic value of tumor-associated autoantibodies panel in combination with traditional tumor markers for lung cancer

**DOI:** 10.3389/fonc.2023.1022331

**Published:** 2023-02-15

**Authors:** Yu Xu, Wenjing Zhang, Tingting Xia, Yuliang Liu, Zhoukui Bi, Liang Guo, Weijia Xie, Ying Xiang, Zhi Xu, Zubin Yu, Yafei Li, Li Bai

**Affiliations:** ^1^ Department of Respiratory and Critical Care Medicine, Xinqiao Hospital, Army Medical University (Third Military Medical University), Chongqing, China; ^2^ Department of Epidemiology, College of Preventive Medicine, Army Medical University (Third Military Medical University), Chongqing, China; ^3^ Department of Respiratory and Critical Care Medicine, the First Affiliated Hospital of Chongqing Medical University, Chongqing, China; ^4^ Department of Thoracic Surgery, North-Kuanren General Hospital, Chongqing, China; ^5^ Department of Thoracic Surgery, Xinqiao Hospital, Army Medical University (Third Military Medical University), Chongqing, China

**Keywords:** lung cancer, tumor-associated autoantibodies, biomarkers, tumor-associated antigens, early diagnostic value

## Abstract

**Introduction:**

The diagnostic value of 7 tumor-associated autoantibodies (AABs) including p53, PGP9.5, SOX2, GAGE7, GBU4-5, MEGEA1, and CAGE for the detection of lung cancer has shown inconsistency in several studies. This study aimed to confirm the diagnostic value of 7AABs and to explore whether the diagnostic value would be improved by combining them with 7 traditional tumor-associated antigens (CEA, NSE, CA125, SCC, CA15-3, pro-GRP, and CYFRA21-1) in clinical settings.

**Methods:**

The plasma levels of 7-AABs were detected by enzyme-linked immunosorbent assay (ELISA) in 533 lung cancer cases and 454 controls. The 7 tumor antigens (7-TAs) were measured by Electrochemiluminescence immunoassay with Cobas 6000 (Roche, Basel, Switzerland).

**Results:**

The positive rate of 7-AABs in the lung cancer group (64.00%) was significantly higher than that of healthy controls (47.90%). The 7-AABs panel was able to discriminate lung cancer from controls with a specificity of 51.50%. After combining the 7-AABs with 7-TAs, the sensitivity showed a significantly enhancement compared with 7AABs panel alone (92.09% vs 63.21%). In patients with resectable lung cancer, the combination of 7-AABs and 7-TAs improved the sensitivity from 63.52% to 97.42%

**Discussion:**

In conclusion, our study found that the diagnostic value of 7-AABs was enhanced when combined with 7-TAs. This combined panel could be used as promising biomarker to detect resectable lung cancer in clinical settings.

## Introduction

Lung cancer remained the leading cause of malignancy cancer death worldwide, with an estimated 1.8 million new cases and an average 5-year survival rate of 17.4% in 2021 (1). Despite the recent advent of promising new targeted therapies, lung cancer diagnostic strategies still have difficulties in identifying the disease at an early stage. The lack of effective strategy for early lung cancer detection accounts for the overall poor prognosis [5-year survival rate of 5.2% for metastasized lung cancer versus 92% for early-stage IA lung cancer (2, 3)]. Therefore, early detection of lung cancer could potentially lead to significant decreases in morbidity and mortality. Low Dosage Computerized Tomography (LDCT) has been shown to improve early detection of lung cancer and reduced mortality rates in high-risk individuals (4), however, its high false-positive rate (50%), repeated radiation exposure limit, and low participation rate has restricted its general application in clinical diagnosis (5). These limitations have inspired sustained interest in identification of valid biomarkers detectable in human plasma. Hence, there is a need for an accurate, non-invasive test that has long been desired to assist the early diagnosis of lung cancer.

Blood-based biomarkers assay for the detection of lung cancer at early stage could be of great help to aid screening. The traditional serum protein biomarkers such as carcinoembryonic antigen (CEA), cytokeratin 19 fragment (CYFRA 21-1), neuron-specific enolase(NSE), Pro-gastrin releasing peptide (ProGRP), carbohydrate antigen (CA)125, and CA19-9 has been used in the clinical routine examination of tumors. However, the sensitivity and specificity of these conventional biomarkers have been limited due to the low number of tumor cells in the serum (<10^6^) in early-stage lung cancer (6).

Many studies demonstrated that tumor-associated antigens (TAAs) were formed during the autologous cells developing into tumors (7). Although only trace amounts of TAAs are present in the blood in early-stage lung cancer, these TAAs can be captured by the immune system and lead to the formation of a large amount of specific tumor-associated autoantibodies (AABs) (8). Autoantibodies can be detected in peripheral blood of patients with solid tumors up to 3–4 years before the onset of symptoms and can therefore be used as a blood-based diagnostic biomarker (9).

Autoantibodies produced by humoral immune response to tumor-associated antigens (TAA) are emerging as promising biomarkers for the non-invasive diagnosis of patients with lung cancer (10). However, due to the complexity of the immune system and tumor heterogeneity, single AAB markers had shown low diagnostic sensitivity in the application of early-stage lung cancer diagnosis (11). A synergistic approach consisting of multiple AABs may provide a new idea for improving the sensitivity. A combination of 6 AABs (p53, NY-ESO-1, CAGE, GBU4-5, Annexin1, and SOX2), known as *Early* CDT-Lung, showed high specificity (89%) and sensitivity of 43% for early-stage lung cancer (12). *Early* CDT-Lung has already been used as a complementary method for the early diagnosis of patients with lung cancer in the US through the Clinical Laboratory Improvement Amendments (CLIA) (13). Considering ethnic differences, Ren et al. identified and validated the clinical value of a 7-AABs panel (p53, PGP9.5, SOX2, CAGE7, GBU4-5, MAGEA1, and CAGE) for the early detection of lung cancer patients in Chinese populations (14). However, this 7-AABs panel for the detection of lung cancer has shown inconsistency in several studies. In this study, we aimed to validate the diagnostic efficiency of the 7-AABs panel in the detection of lung cancer. In addition, we further explored whether 7-AABs combined with traditional biomarkers can improve the diagnostic value of early lung cancer detection.

## Materials and methods

### Patients and samples

A total of 987 patients admitted to Xinqiao hospital of the Army Medical University from 2014 to 2017 were enrolled in this study. Fasting plasma samples were obtained from 485 patients with NSCLC (including 291 patients with adenocarcinoma, 180 patients with squamous cell carcinoma, 10 patients with adenosquamous carcinoma, and 4 patients with large cell carcinoma), 48 patients with SCLC, and 454 controls. All Lung cancer patients were diagnosed according to NCCN guidelines (15). The TNM stage of disease was defined according to the 7^th^ edition of the International Association for the Study of Lung Cancer classification system. All lung cancer patients were newly diagnosed and pathologically confirmed by surgical samples or biopsy tissue. Patients who had received surgery, radiotherapy, chemotherapy or targeted drug or had a history of heart, liver, kidney disease, or diabetes were excluded.

The healthy individuals and patients with benign lung diseases were selected as the control group. Healthy controls were recruited from the participants of routine physical examinations during the same period. The benign lung disease group included patients with chronic obstructive pulmonary disease (COPD), pneumonia, pulmonary tuberculosis, and other diseases (pulmonary embolism, bronchiectasis, interstitial lung disease, etc.). Demographic and clinicopathological characteristics of all participants were obtained through a combination of structured questionnaire and medical records. All blood samples were collected after the informed consent of the patients and the study was approved by the ethical committee of the Army Medical University. For each participant, 5ml blood samples were collected and were separated by centrifugation at 4°C and stored in sterile tubes at -80°C within 4 hours of sample collection without repeated freezing and thawing.

### Quantitative analysis of autoantibodies in serum samples and assay cutoffs

In our study, a panel of seven tumor-associated antibodies including (p53, PGP9.5, SOX2, GAGE7, GBU4-5, MAGEA1, and CAGE) were detected by a commercial enzyme-linked immunosorbent assay (ELISA) kit (LC-AAB01, Cancer Probe Biological Technology Co. Ltd, Hangzhou, China). The experimental procedure was performed according to the manufacture’s instruction: 50μl of diluted serum samples were added to wells coated with antigen and incubated at room temperature for 1 hour. After washing 3 times, 50μl of diluted secondary antibody HRP-conjugated goat anti-human IgG was added. The substrate was added and the color development reaction was terminated after 15 min with 50μl stop solution. Absorbance was measured at 450 nm for the optical density (O.D.) using a microplate reader. The concentration of reference units (RU) of TAABs was calculated by comparison with the standard curve. All samples were assayed simultaneously. Each sample was tested in duplicate. Sample characteristics were blinded to the test performers during and after the autoantibody panel testing.

The selection of the suitable cut-offs for each ABB was adjusted by combining the manufacturer’s recommendations with the distribution of the specific antibody population level. The seven autoantibodies’ positive reference value were as follows: P53 ≥ 13.1U/mL, PGP9.5 ≥ 11.1U/mL, SOX2 ≥ 12.2U/mL, GAGE7 ≥ 14.4U/mL, CBU4-5 ≥ 8.7U/mL, MAGEA1 ≥ 12.3U/mL and CAGE ≥ 7.2U/mL. A positive test result was assigned to the individual if any one of the 7 antibodies levels was above the cut-off values. A negative test result was assigned to the individual when all antibodies were below the cut-off value.

### Detections of serum tumor antigens level

For each individual, 5ml fasting blood samples were collected, centrifuged at 3500r/m for 10 min and detected immediately or stored at -80°C within 1 week. Serum CEA, NSE,CA125, SCC, CA15-3, pro-GRP and CYFRA21-1 levels were measured with an Architech Alinity system (Abbott Molecular, Abbott Park, IL, USA). Serum NSE levels were measured by electrochemiluminescence immunoassay with Cobas 6000 (Roche, Basel, Switzerland).

### Statistical analysis

Continuous variables were presented as Mean and SD if they were under normal distribution, otherwise, median values and interquartile ranges (25th-75th percentile) were taken. Categorical variables were presented as number (%) and compared by χ^2^ or Fisher’s exact test. Kappa measurement was carried out to assess the consistency. All statistical analyses were performed using SPSS statistical software (version 22.0; IBM Corporation, Armonk, NY) and GraphPad Prism 5.0 software (GraphPad Software Inc., San Diego, CA, USA). The MedCalc (MedCalc Software Ltd, Belgium) were used to calculate the sensitivity, specificity, positive predict value, negative predict value. All tests were 2-sided and *P* value < 0.05 was considered to be statistically significant.

## Results

### Study populations

The demographic and clinical characteristics of the participants were summarized in **Table 1**. A total of 533 patients with lung cancer (including 291 adenocarcinoma, 180 squamous cell carcinoma, 10 adenocarcinoma-squamous cell carcinoma, 4 large cell lung cancer and 48 small cell lung cancer) and 454 controls (including 212 benign lung diseases and 242 healthy controls) were enrolled in our study. Age, histological type, stage, gender, and smoking status were collected. There were 383 and 311 males in the lung cancer and controls group, respectively. The median age was higher in control group than in the lung cancer group (*P*<0.001). The proportion of smokers (including both current and former smokers) was significantly higher in the lung cancer group than in the control group (*P*<0.001).

**Table 1:** Clinical and demographic characteristics of the lung cancer cases and controls.

	Lung cancer (n=533)	Benign lung disease and healthy controls (n=454)	*P* value
**Gender**			0.25
Female	150 (28.10%)	143 (31.5%)
Male	383 (71.90%)	311 (68.5%)	
**Age, year (medium,IQR)**	59 (50-65)	62 (52-68)	<0.001
**Smoking history**			<0.001
Never	180 (33.77%)	227 (50.0%)	
Former/current	353 (66.20%)	227 (50.0%)	
**Histological type**			
Adenocarcinoma	291 (54.60% )	–	
Squamous cell carcinoma	180 (33.80% )	–	
Adenocarcinoma-squamous cell carcinoma	10 (1.90% )	–	
Large cell lung cancer	4 ( 0.70%)	–	
Small cell lung cancer	48 (9.00% )	–	
**Stage**			
I	155 (29.10%)	–	
II	81 (15.20%)	–	
III	88 (16.50%)	–	
IV	209 (39.20%)	–	

Continuous data was presented as median and interquartile range. Discrete data was presented as counts and percentages. Gender and smoking history were tested by chi-square test and age was compared by t test. Bold indicates p < 0.05.

### Comparative analysis of the levels of 7-AABs among the lung cancer and control group

The serum concentration of the 7-AABs (including p53, PGP9.5, SOX2, GAGE7, GBU4-5, MAGEA1 and CAGE) in lung cancer and control groups (healthy control and benign lung disease groups) were evaluated by ELISA. We compared the positive rate of a single marker and combined makers in the lung cancer and control group. Among the 7 tumor-associated autoantibodies, six of them were significantly increased in the lung cancer group compared with controls except for GBU4-5 (*P=*0.873). In patients with benign lung disease, the positive rates of GAGE7 and PGP9.5 were higher than those of healthy controls (*P*<0.001), while the positive rates of the other five antibodies did not differ in the two types of controls (**Table S1**).

### The value of 7-AABs panel in diagnosis of lung cancer

**Figure 1:**
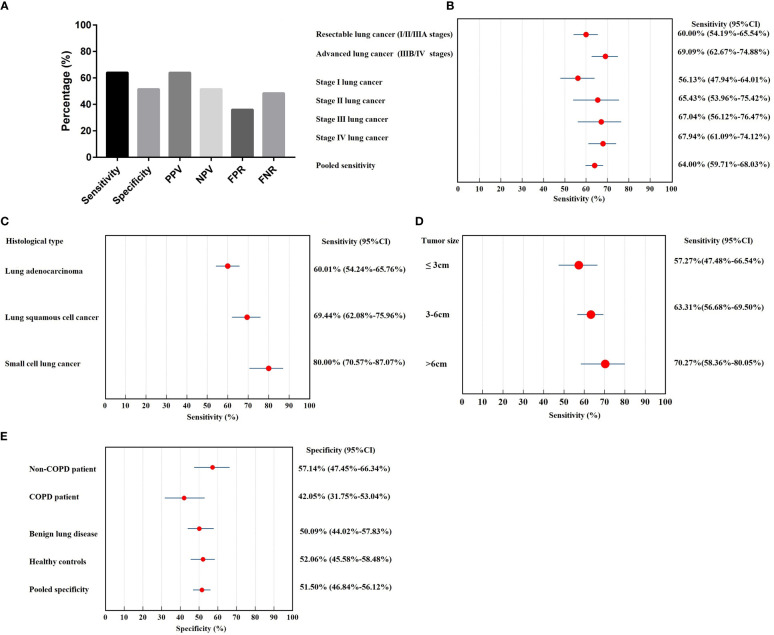
The diagnostic value of 7-AABs panel in lung cancer diagnosis. **(A)** The sensitivity, specificity, PPV, NPV, FPR and FNR of 7-AABs in the diagnosis of lung cancer; **(B)** Sensitivity in subgroups of lung cancer patients stratified by stages; **(C)** Sensitivity in subgroups of lung cancer patients stratified by histological type; **(D)** Sensitivity in subgroups of lung cancer patients stratified by tumor size; **(E)** Specificity in subgroups of controls (healthy control, benign lung diseases, COPD and non-COPD). PPV, positive predictive value; NPV, negative predictive value; FPR, false positive rate; FNR, false negative rate; COPD, chronic obstructive lung disease.

Positivity in the 7-AABs panel was defined as a positive signal for any of the 7-AABs detected using the defined cut-offs. Compared with control, the panel showed a sensitivity of 64.00% and specificity of 51.50%. The positive predictive value (PPV) of 7-AABs was 63.97% and the negative predictive value (NPV) was 51.54%. The false positive rate (FPR) for 7-AABs was 36.02% and the false negative rate (FNR) was 48.49% (**Figure 1A**).

We further performed subgroup analysis to investigate the diagnostic value of the 7-AABs in patients with different stages and histological types of lung cancer. The sensitivity of lung cancer patients ranged from 56.1% to 67.9%, with 60.00% (95% CI, 54.19%-65.54%) in resectable lung cancer (I/II/IIIA stages), 69.09% (95% CI, 62.67%-74.88%) in advanced lung cancer (IIIB/IV stages). The sensitivity increases with the tumor stage, respectively were 56.13% (95% CI, 47.94%-64.01%) in stage I, 65.43% (95% CI, 53.96%-75.42%) in stage II, 67.04% (95% CI, 56.12%-76.47%) in stage III and 67.94% (95% CI, 61.09%-74.12%) in stage IV lung cancer (**Figure 1B**).

The sensitivity of lung adenocarcinoma and lung squamous cancer was 60.10% (95% CI, 54.24%-65.76%) and 69.44% (95% CI, 62.08%-75.96%), respectively. Small cell lung cancer showed the highest diagnostic sensitivity of 80.00% (95% CI, 70.57%-87.07%) among histological types (**Figure 1C**).

We further explored the effect of tumor size on sensitivity and found the sensitivity increases with the tumor size. The sensitivity of tumor size>6cm group showed the highest sensitivity with 70.27% (95% CI, 58.36%-80.05%) and followed by tumor size 3-6cm. In tumor ≤3cm group, the sensitivity was 57.27% (95% CI, 47.48%-66.54%) (**Figure 1D**).

The specificity was not significant in healthy control (52.06%; 95% CI, 45.58%-58.48%) and patients with benign lung diseases (50.90%; 95% CI, 44.02%-57.83%) (**Figure 1E**); the false positive rate was 47.90% in healthy controls and 49.10% in patients with benign lung diseases. Since COPD is an independent risk factor for lung cancer, we further divided patients with benign lung diseases into COPD (n=88) and non-COPD group (n=95). The specificity of COPD patients (42.05%; 95% CI, 31.75%-53.04%) was significantly lower than that of the non-COPD benign lung disease group (57.14%; 95% CI, 47.45%-66.34%) (*P*<0.05, **Figure 1E**).

### Diagnostic value of the 7-AABs panel combination with traditional tumor markers in patients with lung cancer

The tumor-associated antigens (TAAs) including CEA, NSE, CA125, SCC, CA15-3, pro-GRP, and CYFRA21-1 are widely used blood-based biomarkers that help to diagnose lung cancer. We combined the 7-TAs and the positivity was defined as elevated TAs in the 7-TAs panel. In this study, 405 of 533 lung cancer patients had available 7-TAs data, which was used to analyze the efficacy of the combination of 7-TAs with 7-AABs in the diagnosis of lung cancer. We compared the consistency of 7-TAs and 7-AABs in the diagnosis of lung cancer using Kappa measurement, which showed the reactivity of 7-TAs and 7-AABs were not consistent in lung cancer (*P*=0.070).

**Figure 2:**
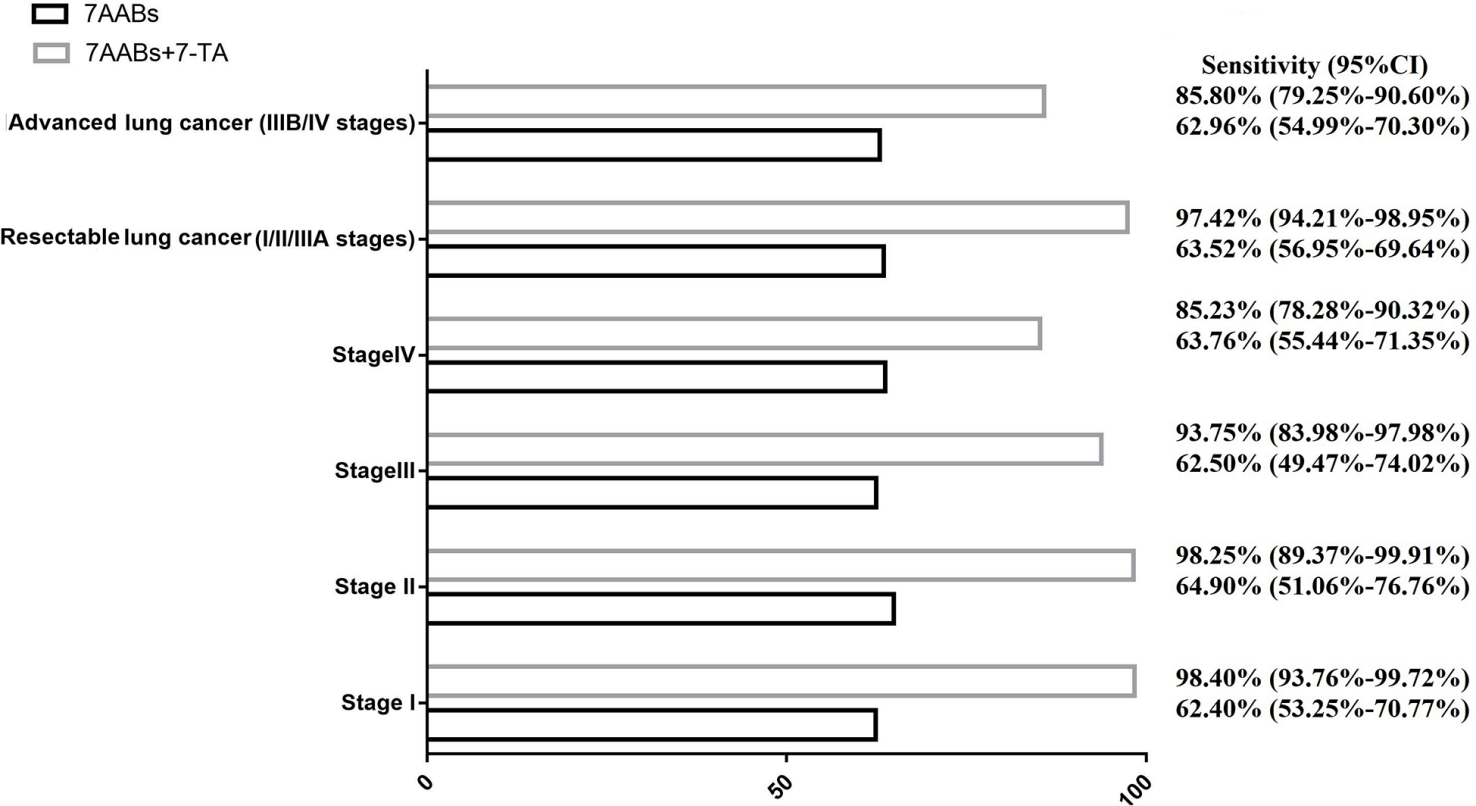
The combined sensitivity of 7-TAs and 7-AABs in diagnosis of lung cancer with different stage.

The inconsistency in the reactivity of 7-TAs and 7-AABs in lung cancer raises the possibility of combining a panel of 7-AABs and 7-TAs to improve the sensitivity of lung cancer diagnosis. We defined a positive result when either of the 7-AABs or 7-TAs assay was positive. Our results showed a significantly higher sensitivity in the combined group compared with 7-AABs panel alone (92.09% vs 63.21%; *P*< 0.001). The improvement in sensitivity was the most significant in stage I lung cancer patients, from 62.40% (95% CI, 53.25%-70.77%) to 98.40% (95% CI, 93.76%-99.72%). In patients with resectable lung cancer, the combination of 7-AABs and 7-TAs improved the sensitivity from 63.52% (95% CI, 56.95%-69.64%) to 97.42% (95% CI, 94.21%-98.95%) (*P*<0.001) (**Figure 2**). Sensitivity of combined diagnosis of 7-AABs and 7-TAs for patients with advanced lung cancer (IIIB/IV stages) improved from 62.96% (95% CI, 54.99%-70.30%) to 85.80% (95% CI, 79.25%-90.60%).

## Discussion

Early diagnosis of patients with lung cancer in the asymptomatic period remains a challenge in clinical practice. Currently, various blood biomarkers have been applied in clinical work of the early screening of lung cancer for the convenience of the patients and minimizing the exposure to radiation. In this study, a panel of seven autoantibodies (including p53, PGP9.5, SOX2, GAGE7, GBU4-5, MAGEA1, and CAGE) were measured in the plasma of lung cancer patients and healthy controls to test the diagnostic value. Subsequently, we performed a combined sensitivity study of 7-AABs with traditional 7-TAs in the diagnosis of lung cancer. The study showed that the 7-AABs panel had clinical value in distinguishing lung cancer patients from healthy controls and benign lung diseases. Combining of 7-AABs with 7-TAs significantly improved the sensitivity of 7-AABs, especially in resectable lung cancer.

Detectable autoantibodies against tumor antigens appeared earlier compared to tumor antigens (16). The value of 7-AABs in the screening and diagnosis of early lung cancer has been recently reported in studies. Ren, et al. have investigated the levels of 7-AABs in patients with lung cancer and analyzed the diagnostic accuracy of 7-AABs alone and in combination (14). Their results indicated that the positive rate of AABs including p53 and GAGE7 in lung cancer patients was not significantly different from that of healthy control, whereas we found no difference between GBU4-5 in lung cancers from controls (healthy control and benign lung disease control). Although the individual AABs showed low diagnostic sensitivity, the combination of 7-AABs provided relatively high sensitivity (64.00%) in our study, which was consistent with that reported by Ren, et al. (61%) (14).

As reported in the Ren et. al., 7-AABs could distinguish lung cancer patients from healthy control with a specificity of 90.00%. Unexpectedly, a large proportion of patients with benign lung diseases were found to respond to 7-AABs in our study, whereas the study of Ren, et al. showed little differences between healthy controls (89%) and benign lung disease (91%). The possible explanation might be that, in our study, the majority of participants with benign lung disease were COPD patients. Subgroup analysis showed the specificity was significantly lower in benign lung disease subgroup with COPD than the non-COPD benign lung disease subgroup.

There are several reasons for the elevated 7-AABs in COPD patients: (1) It has been found that COPD patients produce a variety of auto-antibodies (17). (2) Our previous study found similar immunity profiles of Th17 T cell in lung cancer patients and COPD-lung cancer, indicating a potential inner connection between COPD and lung cancer (18). (3) It has also been reported that the autoantibodies in the circulation can diagnose lung cancer 5 years before CT (19). Patients with COPD have elevated levels of serum 7-AABs may develop lung cancer in the future. It would be interesting to follow up on COPD patients who present 7-AABs positive results and study their risk of developing lung cancer in the future.

The combination of 7-AABs with chest CT has been reported to improve the diagnostic efficacy of 7-AABs and to help in the diagnosis of lung cancer in patients with radiological nodules or shadows on a chest CT (14). However, in clinical settings, the serological assay was usually performed before chest CT to screen high-risk individuals. For the benefit of the patients, it is important to improve the diagnostic efficacy of 7-AABs with other serological markers. The combination of tumor antigens and antibodies have been reported in PAULA’s test, a panel of 3 serum proteins (CEA, CYFRA21-1, and CA125) and 1 autoantibody (NY-ESO-1 AAB) was used to assist with the detection of lung cancer with 74% sensitivity, 80% specificity, and 0.81 AUC, respectively (20). In this study, we combined 7-AABs with 7 traditional tumor antigens in blood samples. Combing 7-AABs with 7-TAs significantly improved the sensitivity of 7-AABs in the diagnosis of lung cancer in all stages. However, the specificity of combined 7-AABs and 7-TAs could not be obtained due to the paucity of data on 7-TAs in control groups.

There are several limitations in our study. First, both cases and controls were selected from a tertiary medical center, which may introduce selection bias. Second, the specificity of 7-AABs in our study was not as high as reported, which might be due to the heterogeneity of the participants and the detection platform in different studies. Finally, the patients were enrolled in this study from 2014 to 2017.To ensure sample quality, we have set up a strict management system to ensure the quality of the blood samples according to the ELISA reagent manufacturer. However, compared with the samples with shorter storage time or fresh samples, we cannot rule out the possibility of partial degradation of antibodies in our samples.

To our knowledge, our study is the first to combine 7-AABs with traditional 7-TA to improve the sensitivity of 7-AABs. Currently, the overuse of chest CT is common in lung cancer early screening in China. Our study could help screen high-risk patients by serologic marker testing before chest CT and therefore to be of clinical significance.

## Data availability statement

The raw data supporting the conclusions of this article will be made available by the authors, without undue reservation.

## Ethics statement

The studies involving human participants were reviewed and approved by the ethics committee of the Army Medical University (Chongqing, China). The patients/participants provided their written informed consent to participate in this study.

## Author contributions

YXu: sample collection, data analysis and writing initial draft. WZ: clinical data collection and data management. TX: data analysis and writing draft. YLL: data analysis. ZB: sample clinical information collection. LG: clinical data collection. WX: data verification and language modification. YXi: data management and analysis. ZX: clinical data collection. ZY: study design, sample collection, patients’ follow-up. YFL: study design, statistical analysis, reversion of the article. LB: study design, administration, funding support and reversion of the article. All authors contributed to the article and approved the submitted version.
