# Supplementary material for: Diagnostic value of tumor-associated autoantibodies panel in combination with traditional tumor markers for lung cancer
Source: Front Oncol. 2023 Feb 15;13:1022331. doi: 10.3389/fonc.2023.1022331 (PMC9975551; doi:10.3389/fonc.2023.1022331)
Supplement: Supplementary file 1 [file Table_1.docx]

**Table S1. The diagnostic efficacy of individual antibody and combined 7-AABs in lung cancer**

| Group | Lung cancer  (n=533) | Control (healthy controls and benign lung diseases n=454) | Healthy control  (n=242) | Benign lung disease  (n=212) | Pa | Pb | Pc | Pd |
| --- | --- | --- | --- | --- | --- | --- | --- | --- |
| **Panel positive (n)** | 341 | 220 | 116 | 104 |  |  |  |  |
| **Individual antibody positive (%)** |  |  |  |  |  |  |  |  |
| p53 | 44  (8.3%) | 18  (4.0%) | 9  (3.70%) | 9  (4.2%) | **0.006** | **0.002** | 0.055 | 0.774 |
| PGP9.5 | 66  (12.4%) | 22  (4.8%) | 5  (2.10%) | 17  (8.00%) | **<0.001** | **<0.001** | 0.088 | **0.004** |
| SOX2 | 206  (38.6%) | 136  (30.0%) | 73  (29.2%) | 63  (29.7%) | **0.004** | **0.023** | **0.022** | 0.917 |
| GAGE7 | 80  (15.0%) | 24  (5.30%) | 5  (2.1%) | 19  (9.0%) | **<0.001** | **<0.001** | **0.028** | **0.001** |
| GBU4-5 | 100  (18.8%) | 87  (19.2%) | 43  (17.8%) | 44  (20.8%) | 0.873 | 0.741 | 0.534 | 0.420 |
| MAGEA1 | 78  (14.6%) | 32  (7.00%) | 16  (6.60%) | 16  (7.50%) | **<0.001** | **0.002** | **0.009** | 0.698 |
| CAGE | 40  (7.50%) | 11  (2.40%) | 5  (2.10%) | 6  (2.80%) | **<0.001** | **0.003** | **0.017** | 0.579 |

^a^ lung cancer group vs control

^b^ lung cancer group vs healthy control

^c^ lung cancer group vs benign lung disease control

^d^ healthy control vs benign lung disease control

The differences in positive rates between groups were compared using the chi-square test.
